# Supplementary material for: Investigation of Aggregation and Disaggregation of Self-Assembling Nano-Sized Clusters Consisting of Individual Iron Oxide Nanoparticles upon Interaction with HEWL Protein Molecules
Source: Nanomaterials (Basel). 2022 Nov 10;12(22):3960. doi: 10.3390/nano12223960 (PMC9696017; doi:10.3390/nano12223960)
Supplement: Supplementary file 1 [file nanomaterials-12-03960-s001.zip › nanomaterials-1954122-supplementary.pdf]

Supplementary materials

Section S1: Materials and Methods

1.1. Animals

All manipulation with animals and fibroblast cell isolation was performed at the Institute of Cell Biophysics, Russian Academy of Sciences (Pushchino, Russia). Male mice of the BALB/c line weighing 22–30 g were used in the study. The animals were purchased from the nursery of laboratory animals "Pushchino" (FIBCh RAS, Pushchino, Russia). All experiments with laboratory animals were carried out by the regulatory legal act of the laboratory public health of the Russian Federation No. 199-n "General cases of application of the rules of good practice, international legal standards for use in appropriate conditions" ETS No. 123 "On the protection of vertebrate animals, and scientific research" and, according to indications, with laboratory animals of the ICB RAS No. 57.30.12.2011. Animals were adapted to conventional conditions and got water and food without restrictions.

1.2. Isolation of fibroblasts from murine lungs

Isolation of fibroblasts from murine lungs was performed according to the standard protocol with minor modifications. Mice were immobilized by dislocation of the neck. The lungs were removed from the chest, placed in a Petri dish with sterile HBSS, and washed replacing the solution. The organs were chopped with sterile scissors to pieces with a volume of ~1 mm^3. The minced organs were incubated for 1 h in 25 ml of DMEM medium containing 0,2% (m/v) collagenase type II at 37 °C. Collagenase was inactivated with 20% FBS. Organs were resuspended by pipetting and the suspension was passed through a 70 μm EASTstrainer™ sieve (Greiner bio-one, Austria). Cells were washed with two centrifugations at 400 g for 5 min in DMEM.

The isolated cells were cultured in TC T-25 flasks (Eppendorf, Germany) in DMEM: F12 medium supplemented with 10% FBS, 2 mM L-glutamine, 100 U/ml penicillin, 100 μg/ml streptomycin (PanEco, Russia).

After reaching 80-90% confluency, the cells were detached using Trypsin 0.05% EDTA solution (PanEco, Russia) for 5 min at 37 °C. The detaching solution was inactivated by 10% FBS. Cells were washed with PBS and suspended in a culture medium to obtain a final concentration of 10^7 cells/ml. Preliminary heated (140 °C, 1,5 h) round coverslips (25 mm diameter) were planted in 6-well plates (1 coverslip/well). Cell suspension (50 μl in each sample) was applied on each coverslip. Plates were incubated for 45 min in a CO_2 incubator for cell adhesion. 1 ml of culture medium was added into each well with a coverslip. Plates were incubated for 24 h in a CO_2 incubator before experiments.

1.3. FTIR Spectrometry

Protein samples were studied on a single-beam FSM-2202 IR-Fourier spectrometer in the range of 400–4000 cm–1. A sample with a protein solution of 90 μl was applied to the middle of the germanium plate 20 × 50 mm. The sample was dried for 20 minutes at room temperature and relative humidity ~25%. The spectrum of the germanium plate was subtracted from the spectrum of the sample. Since the thickness of the formed film after the drying of the solutions was not controlled, the spectra in the ranges of 400-800 cm^{-1}, 1400–1800 cm^{-1}, and 2600–3800 cm^{-1} were normalized to the highest peak for this sample in the appropriate range. The spectra were recorded with a resolution of 2 cm^{-1} and 50 averaging.

Section S2: Results

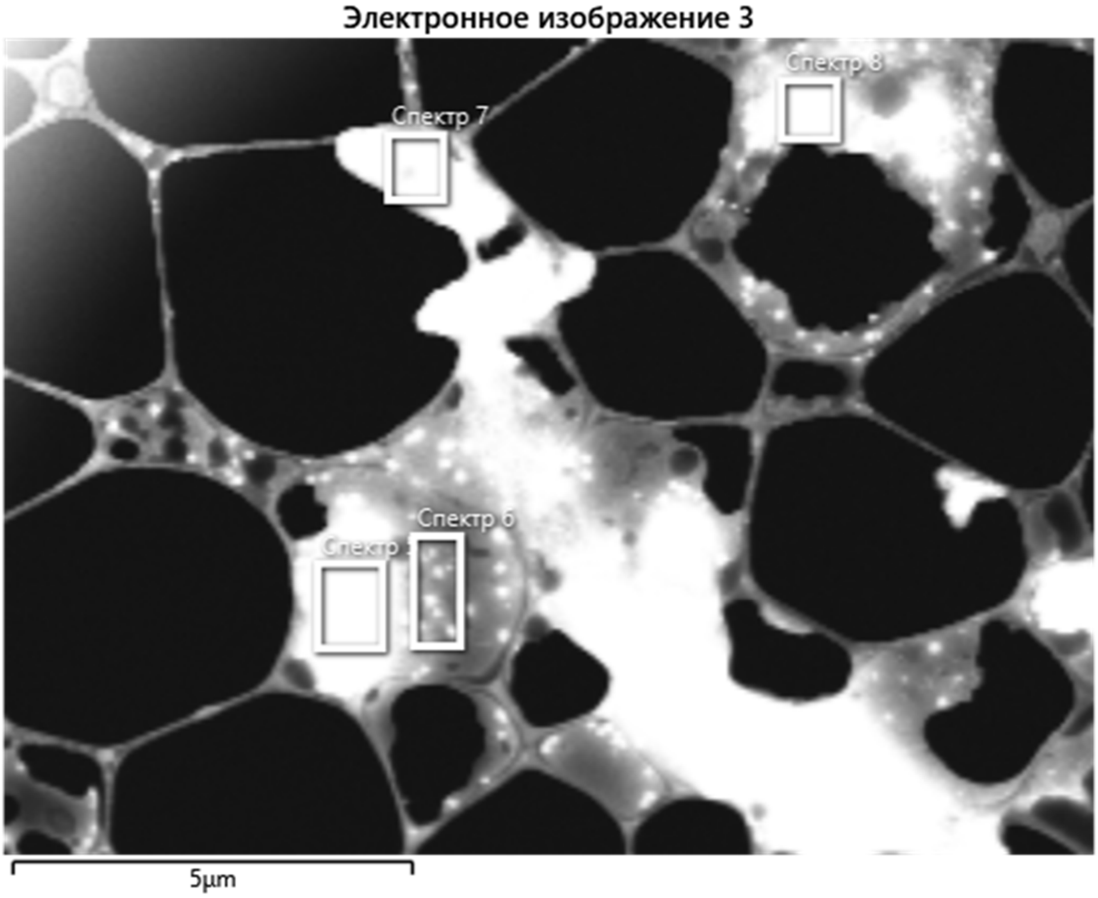

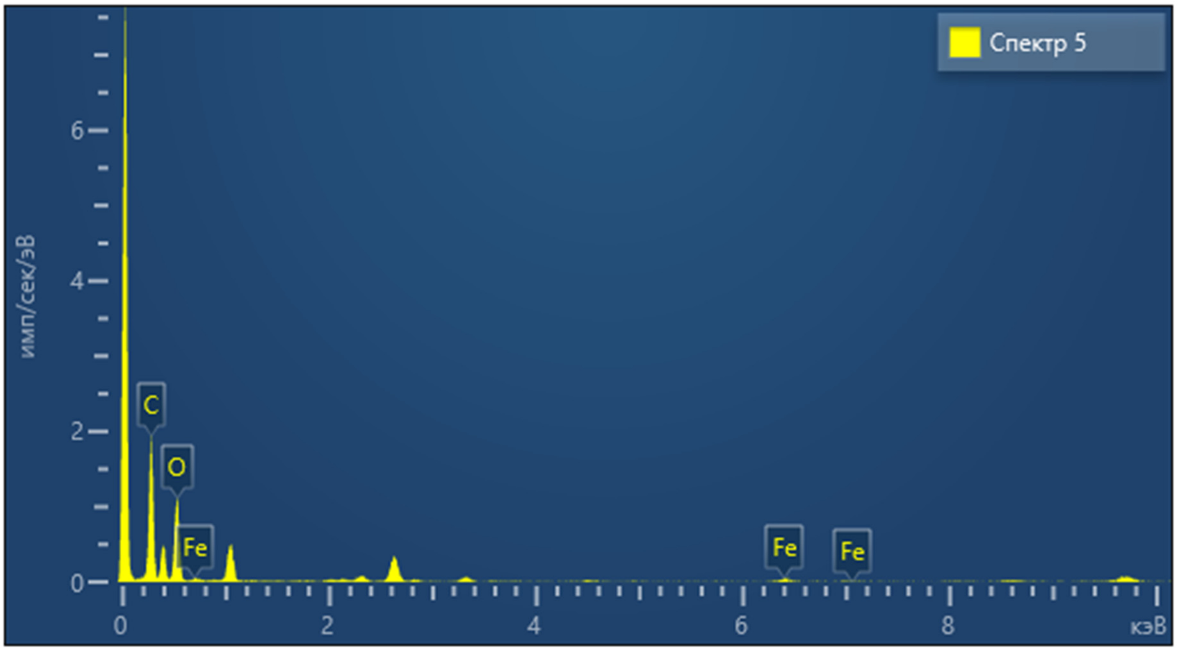

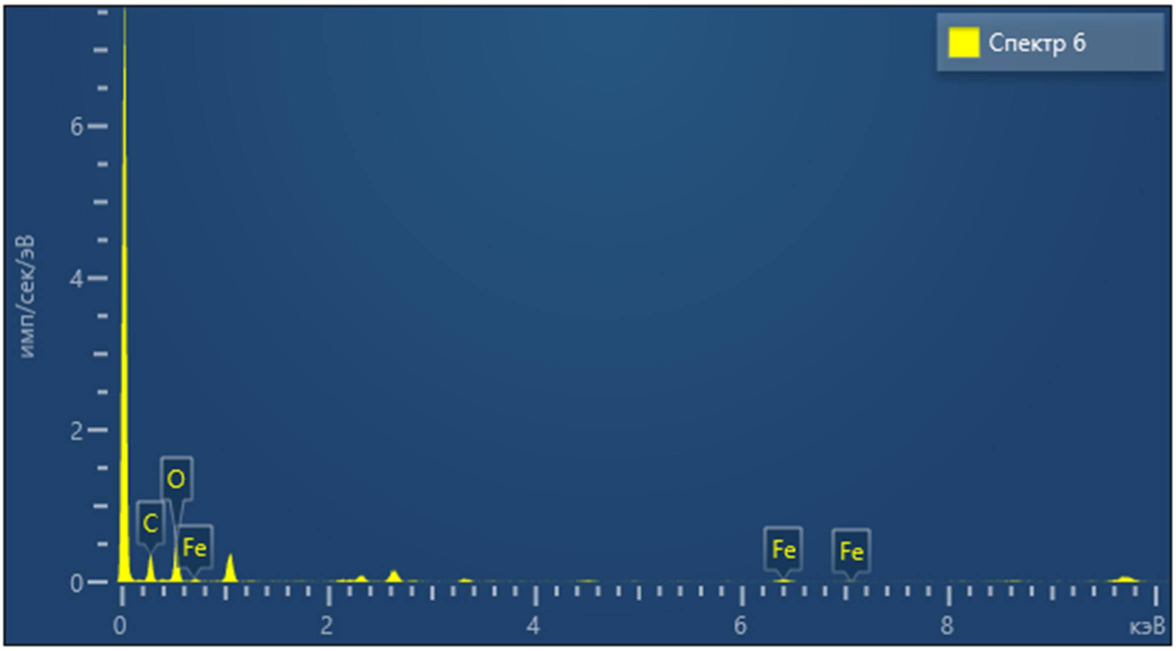
Спектр 6
имп/сек/эВ
0
2
4
6
C
O
Fe
Fe
Fe
8
кэВ

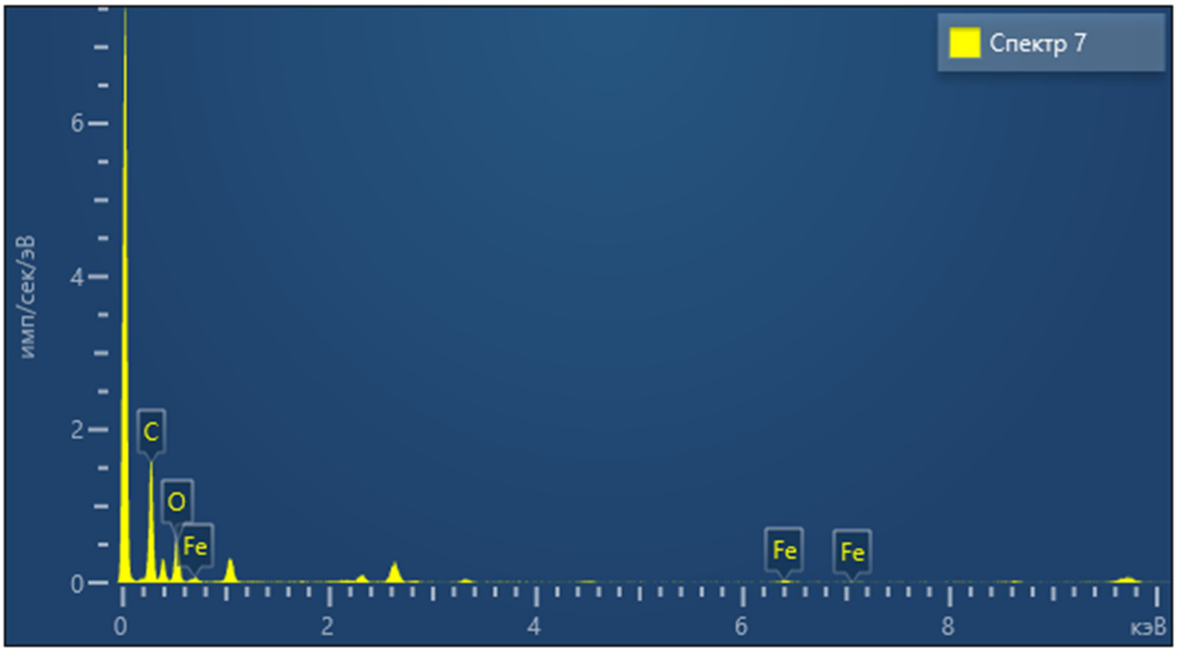
Спектр 7
имп/сек/эВ
0
2
4
6
C
O
Fe
Fe
Fe
8
кэВ

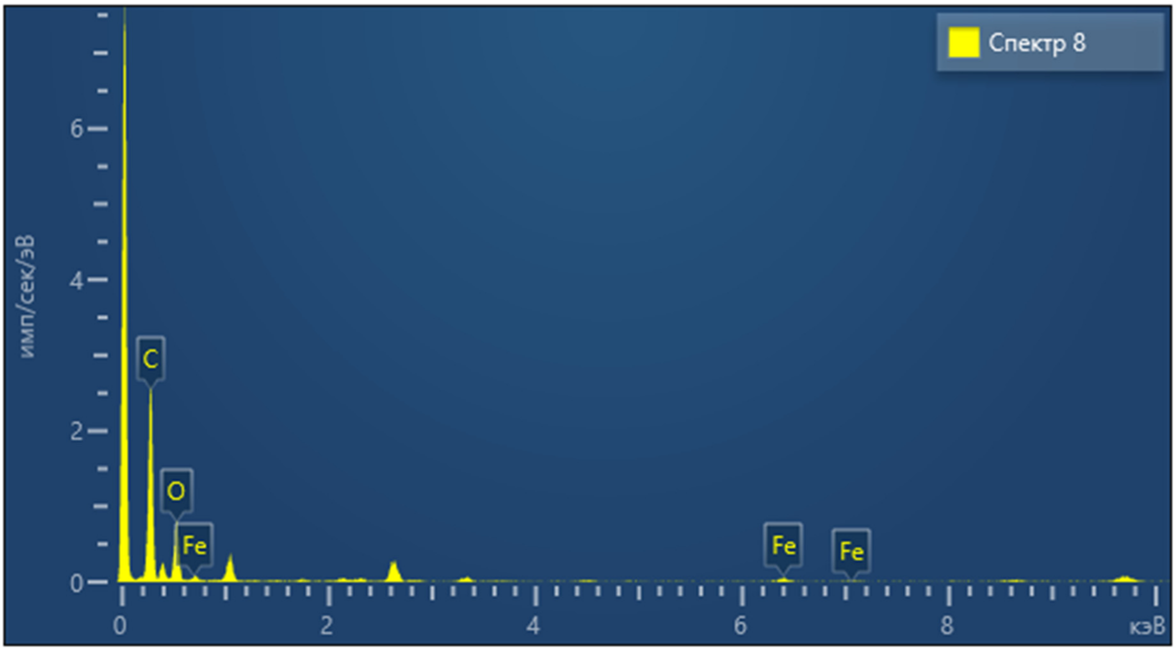

Figure S1a. STEM picture and elemental composition of the sites with TSE-IONPs

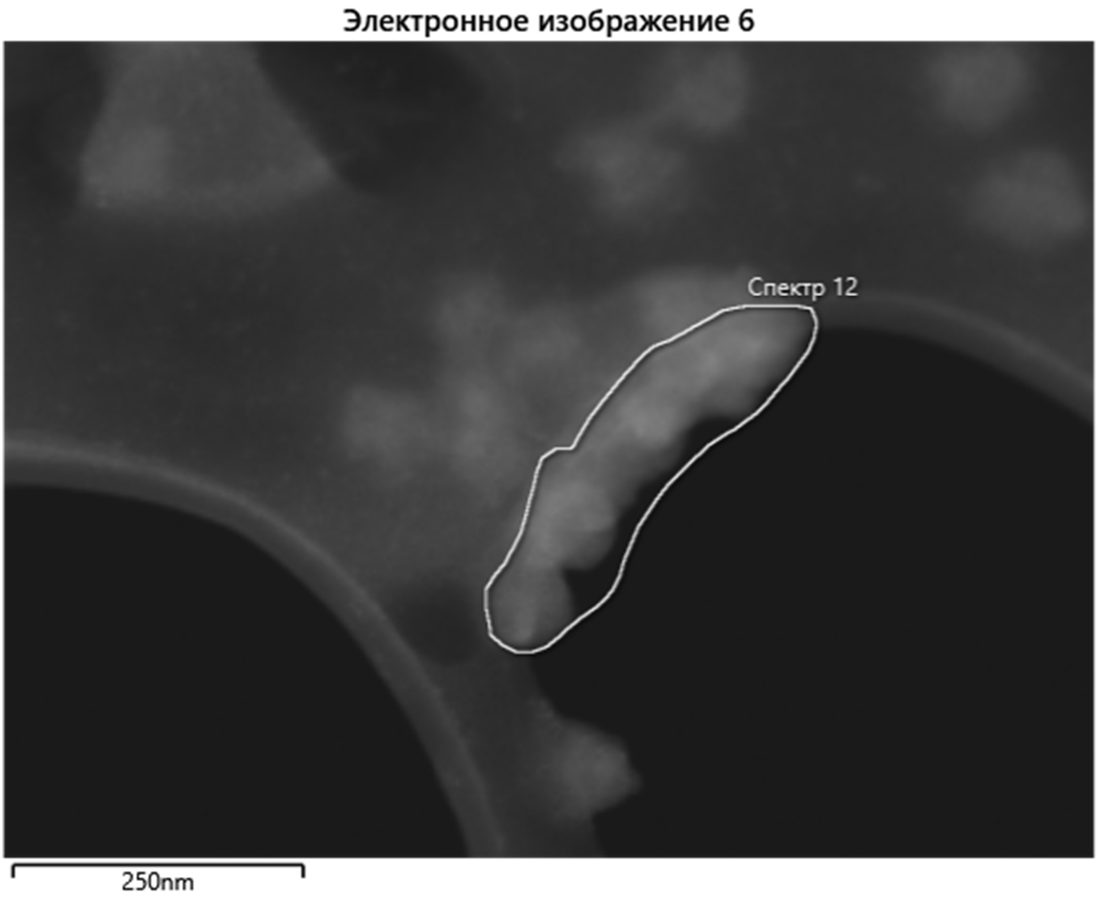

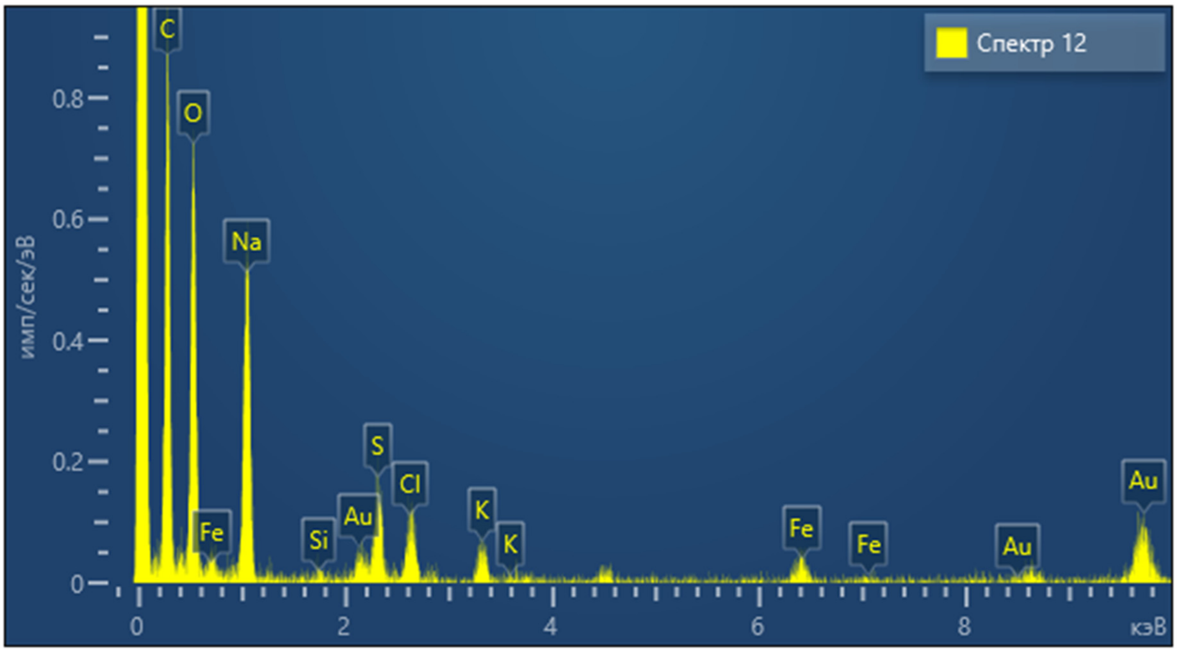

Figure S1b. STEM pictures and elemental composition of the sites HEWL and TSE-IONPs.

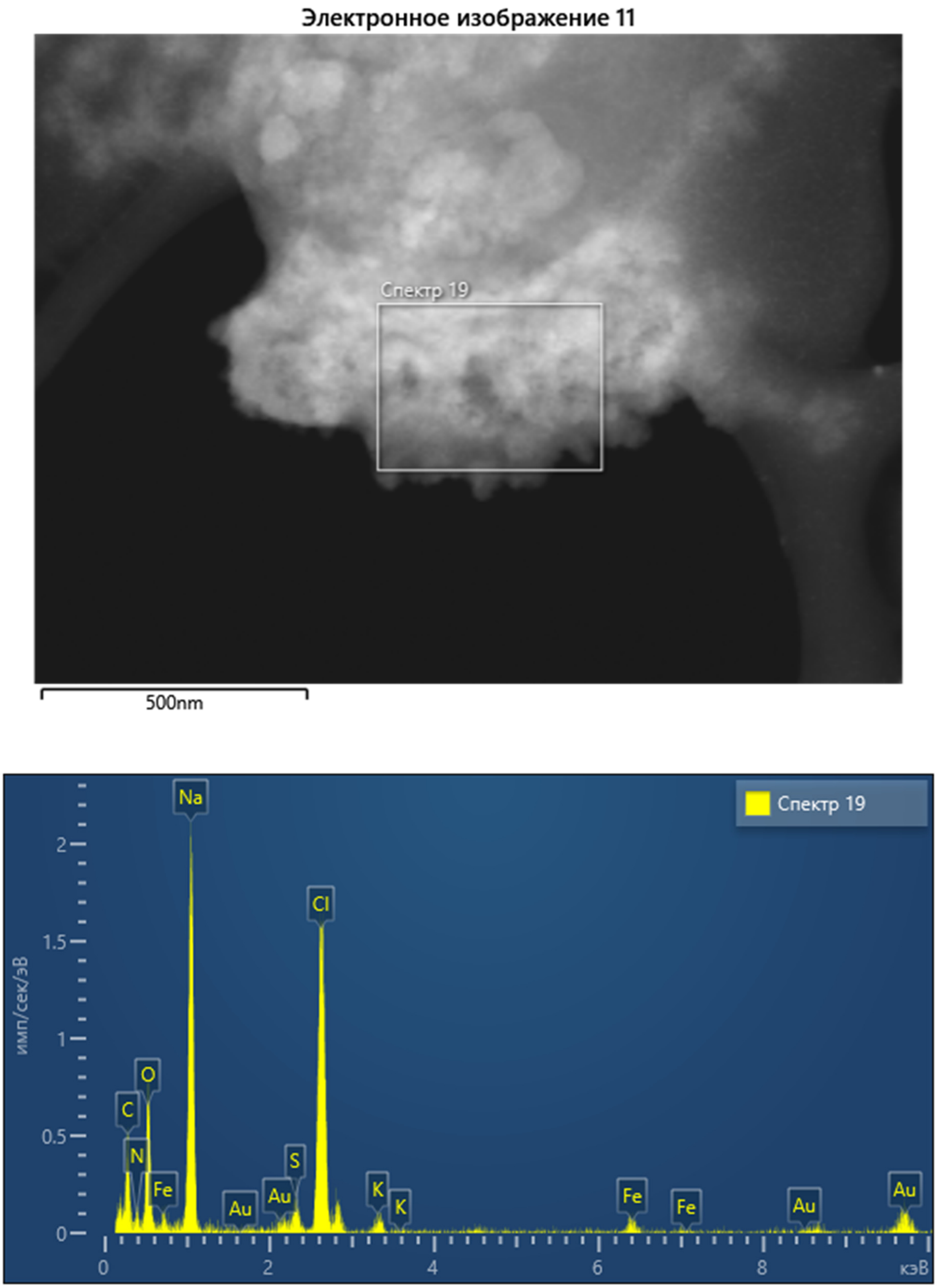

Figure S1c. STEM pictures and elemental composition of the sites HEWL and TSE-IONPs.

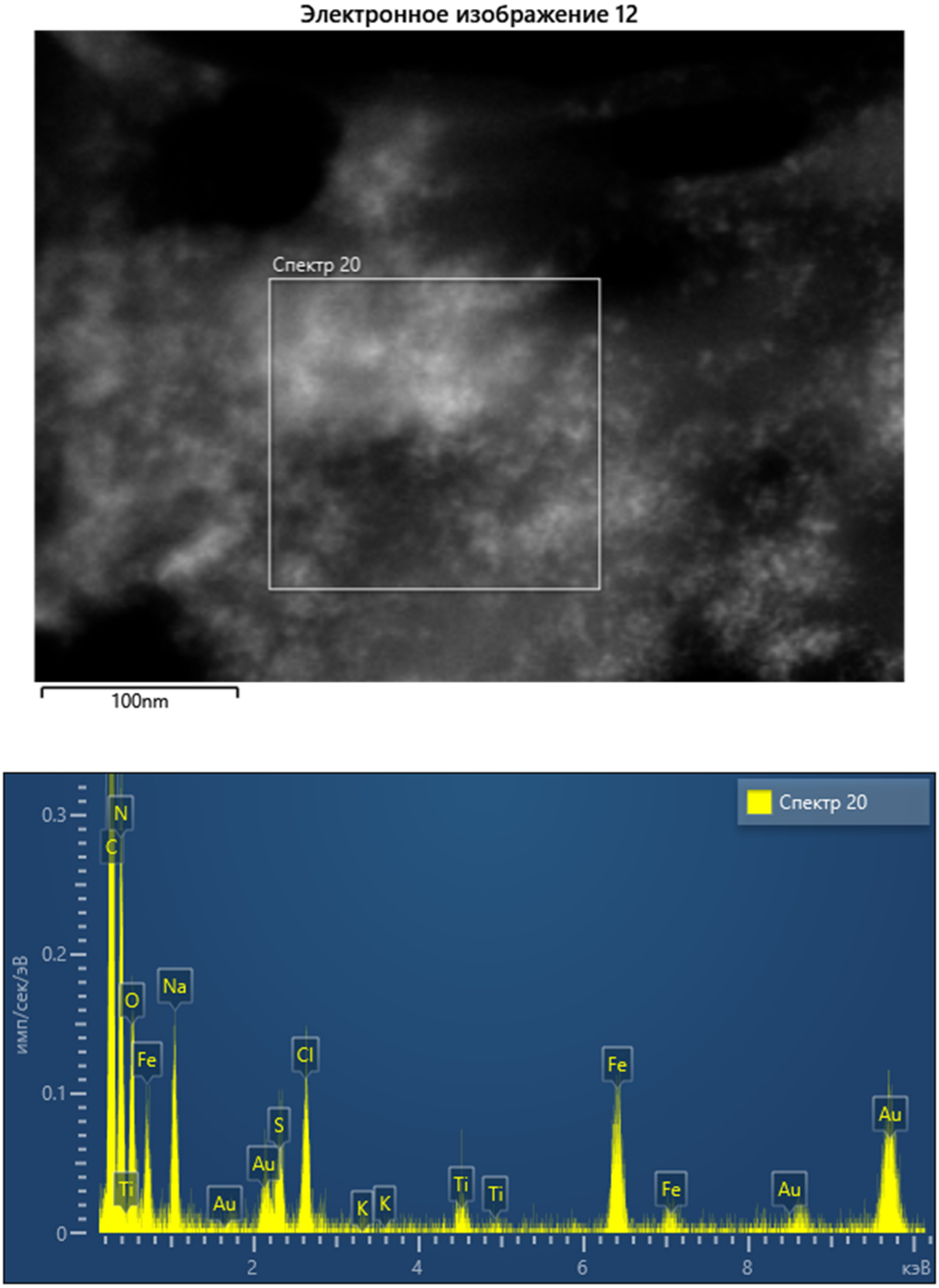

Figure S1d. STEM pictures and elemental composition of the sites HEWL and TSE-IONPs.

Table S1. The elemental composition according to the spectra in figures Y1 and Y2

Samp le	Spectrum Label	O,%	Na,%	Fe,%
TSE-IONPs	"5"	79.36	19.13	1.51
	"6"	66.72	30.62	2.66
	"7"	76.50	21.76	1.74
	"8"	79.01	18.49	2.49
	**Mean**	**75±6**	**23±6**	**2.1±0.6**
TSE-IONPs with HEWL	"12"	67.05	30.98	1.97
	"19"	37.99	60.12	1.89
	"20"	65.38	19.52	15.09
	**Mean**	**57±16**	**37±21**	**6±7**

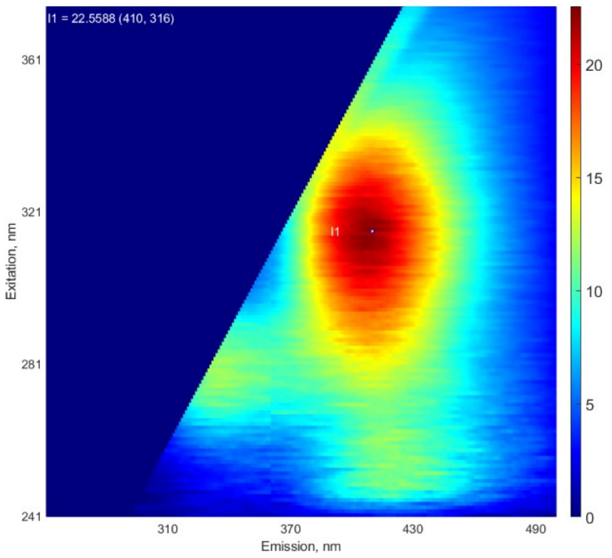

Figure S2. Fluorescence of trisodium citrate 1M. With the same instrument settings as protein fluorescence in the main article.

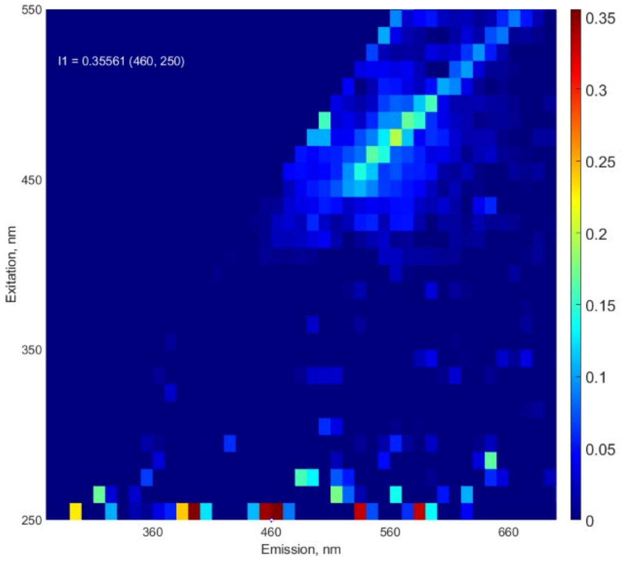

Figure S3. Fluorescence of TSC-IONPs (concentration 10^{13} NPs/ml). With the same instrument settings as protein fluorescence in the main article.

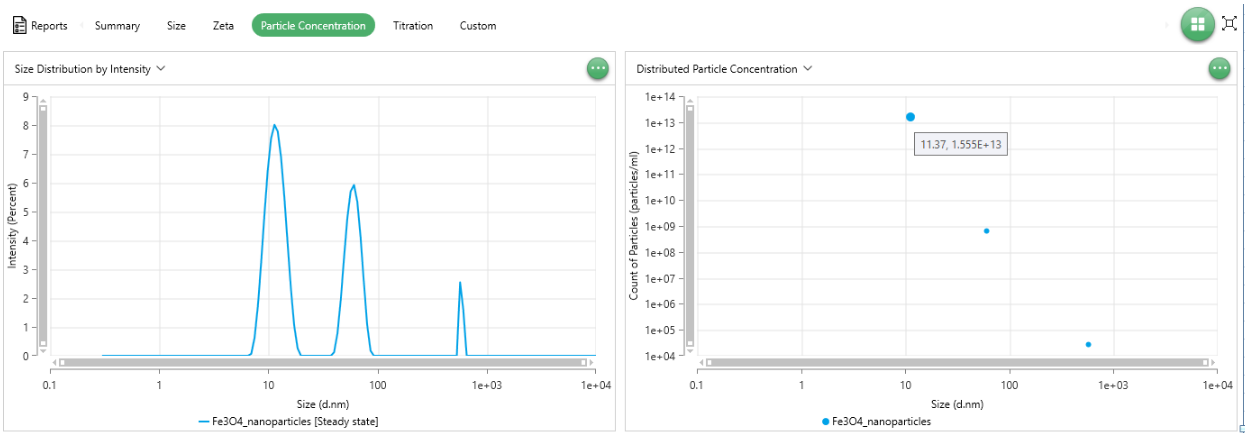

Figure S4. Particle concentration measurements for TSC-IONPs on Zetasizer ULTRA. Here the stock solution was diluted 10^3 times.

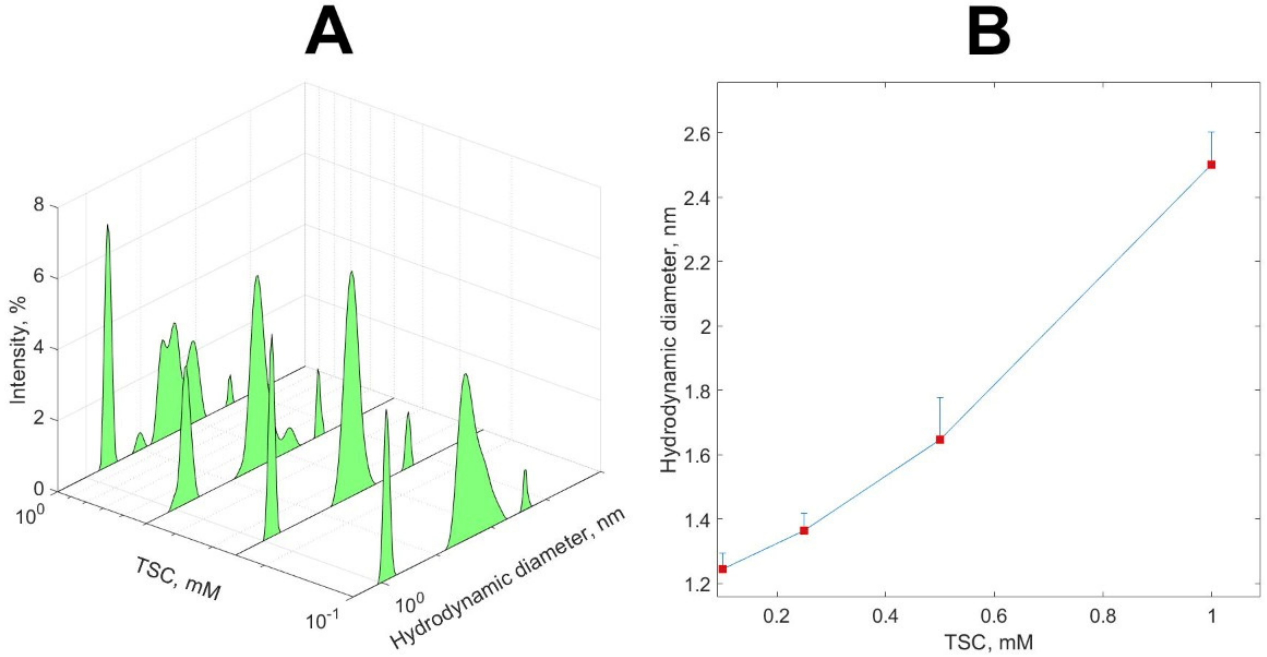

Figure S5. MADLS results. (**A**) Value of peaks intensities and hydrodynamic diameters of the particles in a solution of HEWL (5 mg/ml) with TSC as a concentration function of the trisodium citrate. (**B**) Mean value of the hydrodynamic diameters of the particles of the first peaks in a solution of HEWL with TSC as a concentration function of the trisodium citrate.

Table S2. ζ-potential (mV) and pH for solution HEWL (5 mg/ml) and TSC with different concentrations.

TSC concentration, mM	ζ-potential, mV	pH
0.1	27.7±2.6	4.0±0.1
1	31.4±1.8	5.1±0.1

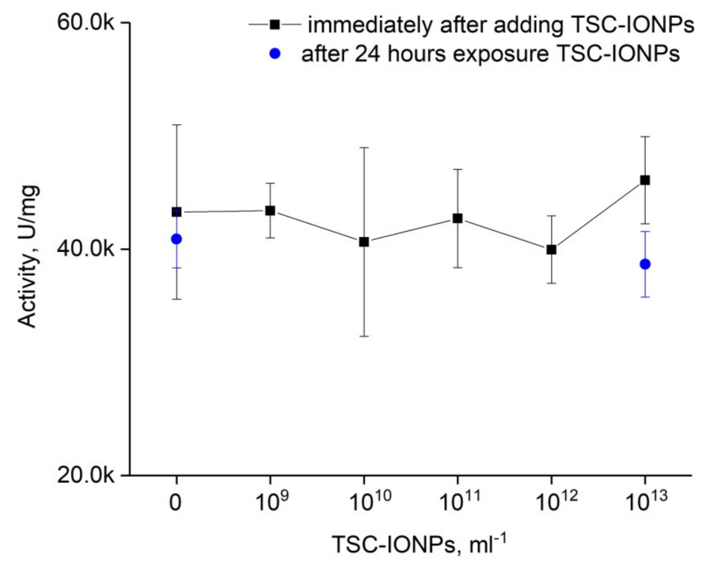

Figure S6. The enzymatic activity of HEWL (5 mg/mL) with different concentrations TSC-IONPs (22 °C) immediately (black square) and after 24 exposure NPs (blue circle). The data are presented as the mean and standard deviation calculated for three test runs.

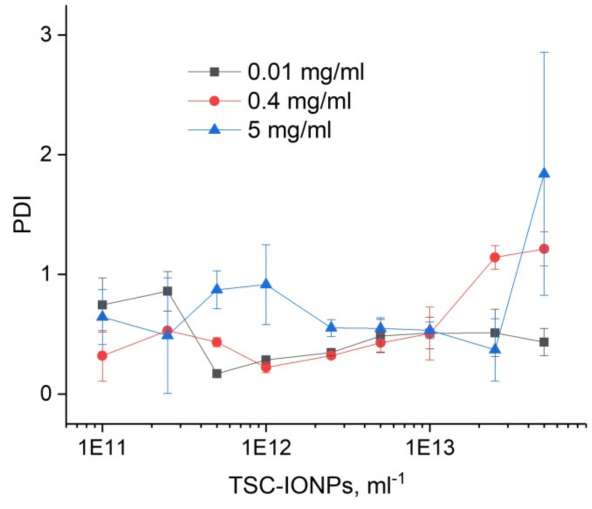

Figure S7 Polydispersity index for MADLS data, figure 6 main article. The data are presented as the mean and standard deviation calculated for three test runs.

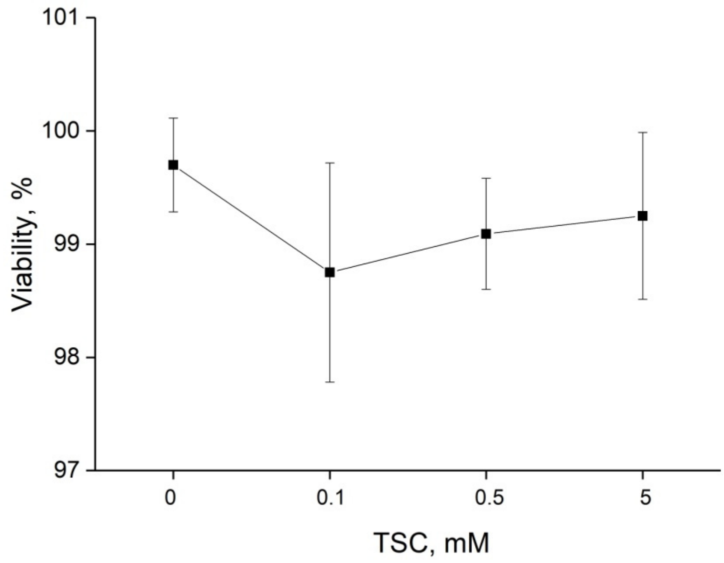

Figure S8. Fibroblasts cell viability after 24 hours of exposure to TSC of various concentrations.

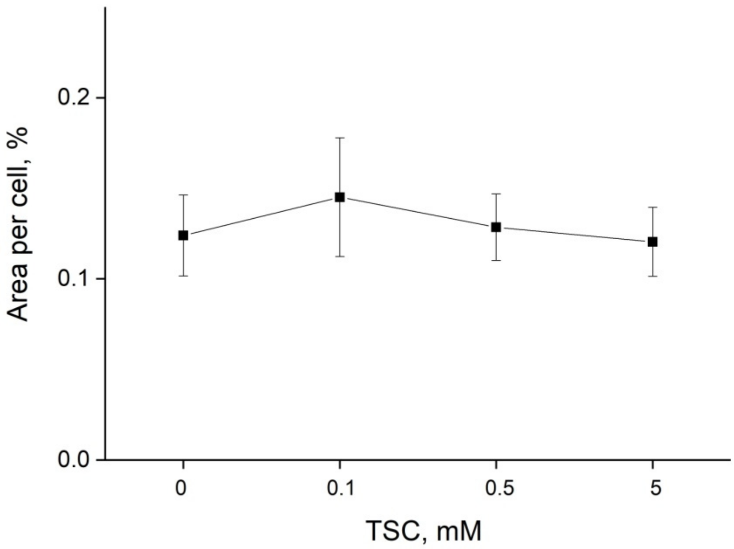

Figure S9 The area occupied by a fibroblast cell relative to the entire frame area after 24 hours of exposure to TSC of various concentrations.

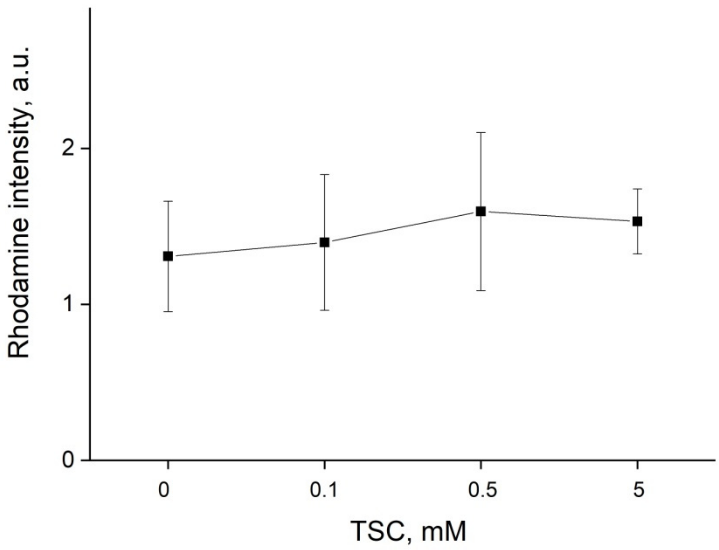

Figure S10 Rhodamin intensity in a fibroblast cell after 24 hours of exposure to TSC of various concentrations.

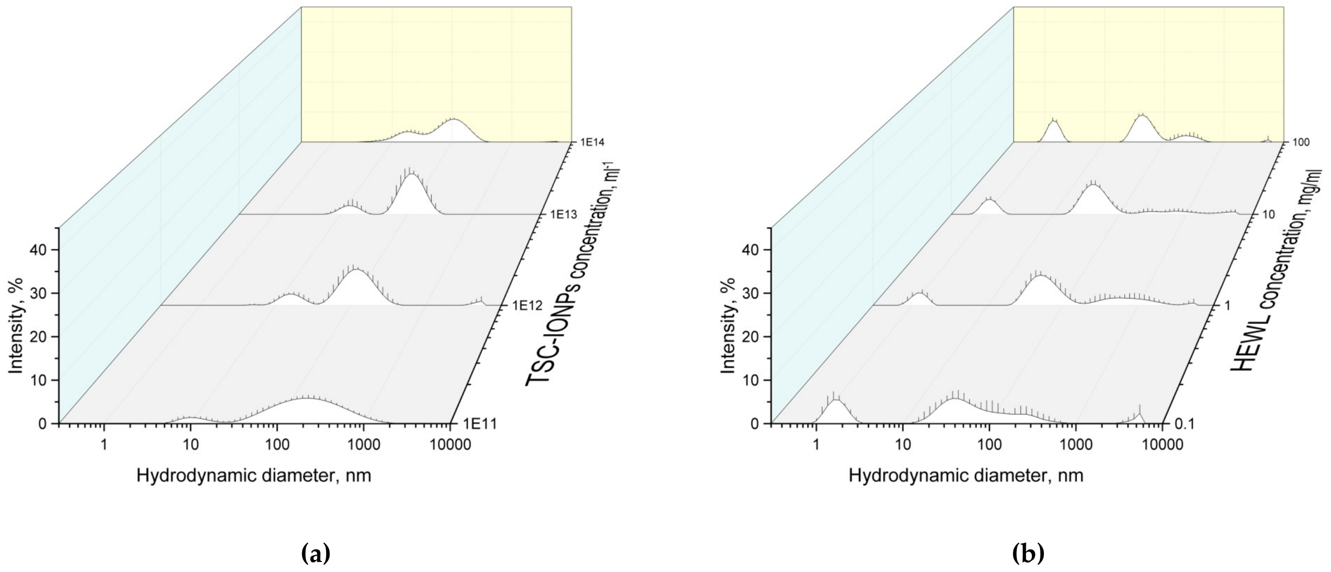

Figure S11. Distribution (weighted by intensity) of hydrodynamic diameters for TSC-IONPs clusters for concentrations from 10^{11} to 10^{14} ml^{-1} (**a**) and molecules and aggregates for HEWL for concentrations from 0.1 to 100 mg/ml (**b**).

Table S3. Settings of the fluorimeter Jasco FP-8300 for Figure 5b–f main article.

Mode	For measurement 0.01, 100 mg/ml HEWL	For measurement 0.4, 5 mg/ml HEWL
Ex bandwidth nm	5	5
Em bandwidth nm	5	1
Response sec	1	1
Sensitivity nm	High	High
Measurement range	280 - 500	280 - 500
Data interval nm	0.2	0.2
Ex wavelength nm	300.0	300.0
Scan speed nm/min	1000	1000
No. of cycles	1	1
Auto gain	Off	Off
Shutter control	Open only for measurement	Open only for measurement
Light source	Xe lamp	Xe lamp
Filter	Stop scan for exchanging filter	Stop scan for exchanging filter
Auto Zero control	On	On
Blank correction	Off	Off

Because the experiments at 0.01 mg/mL (Figure 5 main article) were performed at different settings compared to 0.4 and 5 mg/mL, it was doubtful that these results were due to a change in fluorimeter settings. Additional measurements were taken and the results were added to **Supplementary Materials (see below)**.

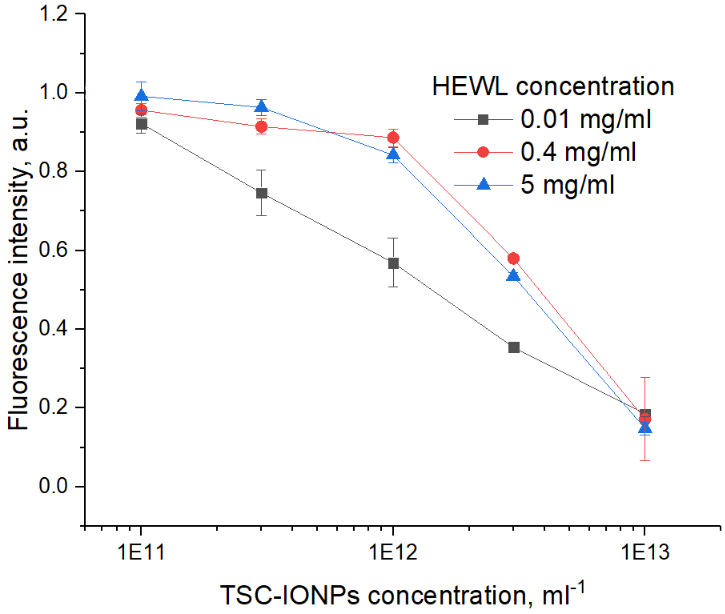

Figure S12. Emission of HEWL samples of various concentrations upon the addition of TSC-IONPs. The samples were excited at 300 nm, the emission peak was at ~337 nm for different concentrations of HEWL. The data are normalized to the maximum intensity of the control protein at the same concentration. Results are presented as mean and SD for three independent experiments. The experiment duplicates the measurements in Figure 5 main paper

The results of an additional experiment (Figure S12) confirmed the previously obtained results (Figure 5b–f main article). Figure S18 shows the initial data on which the point for TSC-IONPs with concentration 10^{12} ml^{-1} was built in the previous Figure S17. Here, all emission curves were taken at the same settings. It can be seen that for a protein concentration of 0.01 mg/ml, the control value of the maximum is too small, and data normalization can lead to an artifact. Next, we took the fluorescence for HEWL 0.01 mg/ml (control and NPs with concentration 10^{12} ml^{-1}, Figure S19) at different settings, so that the peak value in the control was about 1500, approximately, it can be seen that the ratio of control to 10^{12} ml^{-1} did not change.

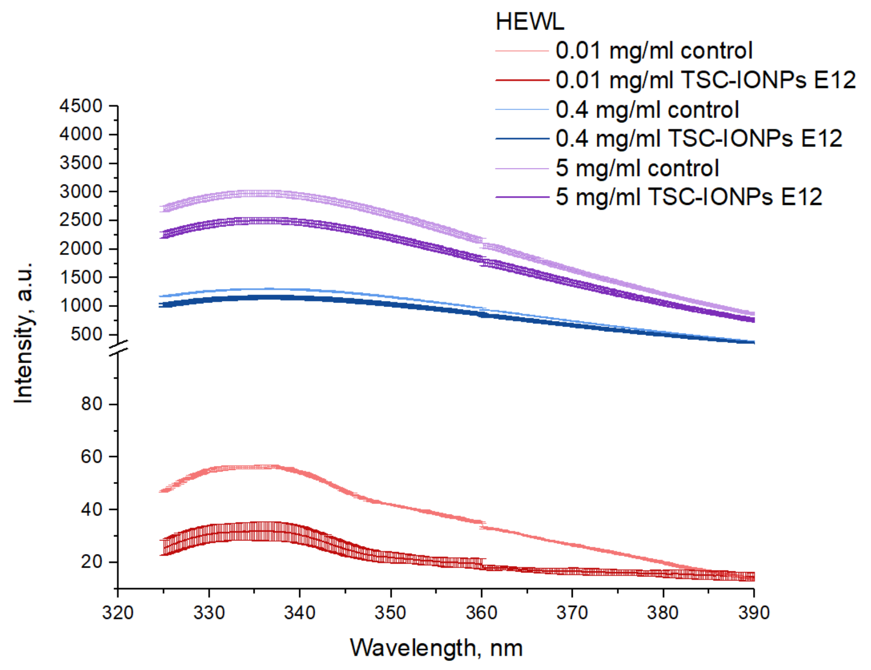

Figure S13. Fluorescent emission of HEWL samples (0.01, 0.4 and 5 mg/ml (b)) for 300 nm excitation for concentrations TSC-IONPs 10^{12} ml^{-1}. Results are presented as mean and standard deviation for three independent experiments.

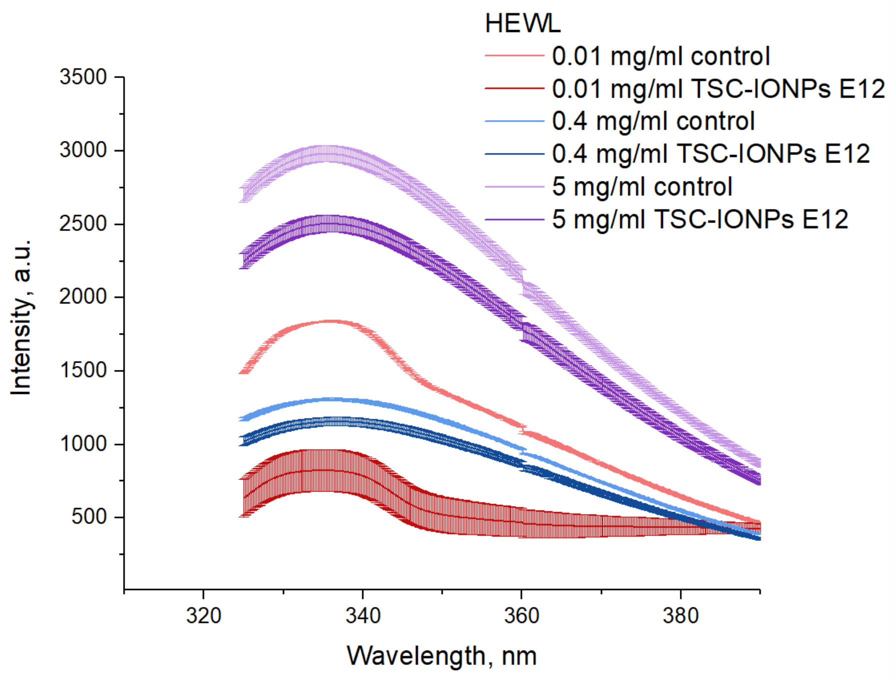

Figure S14. Fluorescent emission of HEWL samples (0.01, 0.4 and 5 mg/ml) for 300 nm excitation for concentrations TSC-IONPs 10^{12} ml^{-1}. Measurements at a concentration of 0.01 mg/ml were performed in other settings (**Table S4**). Results are presented as mean and standard deviation for three independent experiments.

Table S4. Settings of the fluorimeter Jasco FP-8300 for Fig. S12, Fig. S13, Fig. S14

Mode	For measurement 0.01, 0.4, 5 mg/ml HEWL (Fig. S17 and Fig. S18)	For measurement 0.01 mg/ml (Fig. S19)
Ex bandwidth nm	5 nm	5 nm
Em bandwidth nm	1 nm	2.5 nm
Response sec	1 sec	1 sec
Sensitivity nm	High	Manual, 600 V
Measurement range	280 - 390 nm	280 - 390 nm
Data interval nm	0.2 nm	0.2 nm
Ex wavelength nm	300.0 nm	300.0 nm
Scan speed nm/min	1000 nm/min	1000 nm/min
No. of cycles	1	1
Auto gain	Off	Off
Shutter control	Open only for measurement	Open only for measurement
Light source	Xe lamp	Xe lamp
Filter	Filter Stop scan for exchanging filter	Stop scan for exchanging filter
Auto Zero control	On	On
Blank correction	Off	Off

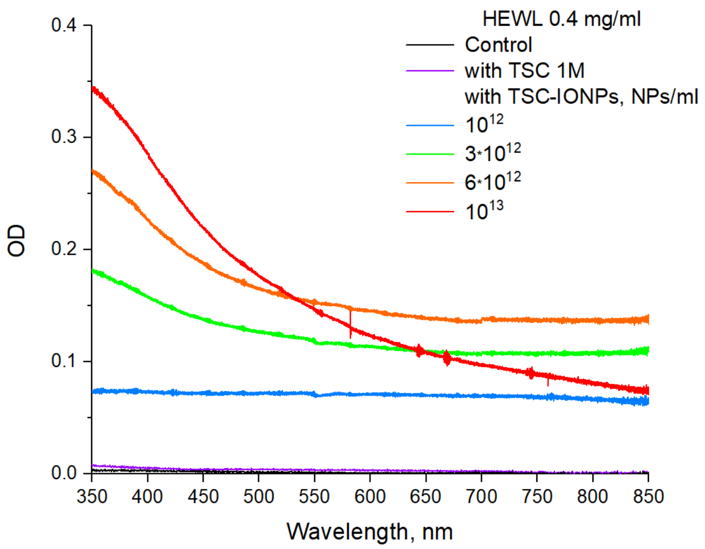

Figure S15. Absorption of HEWL solution (0.4 mg/ml) with the addition of various concentrations of TSC-IONPs (up to 10^{13} ml^{-1} —red line) and TSC 1M (purple line) in 350–850 nm range. Measurements were made relative to water for protein, or relative to the respective concentrations of TSC-IONPs and TSC.

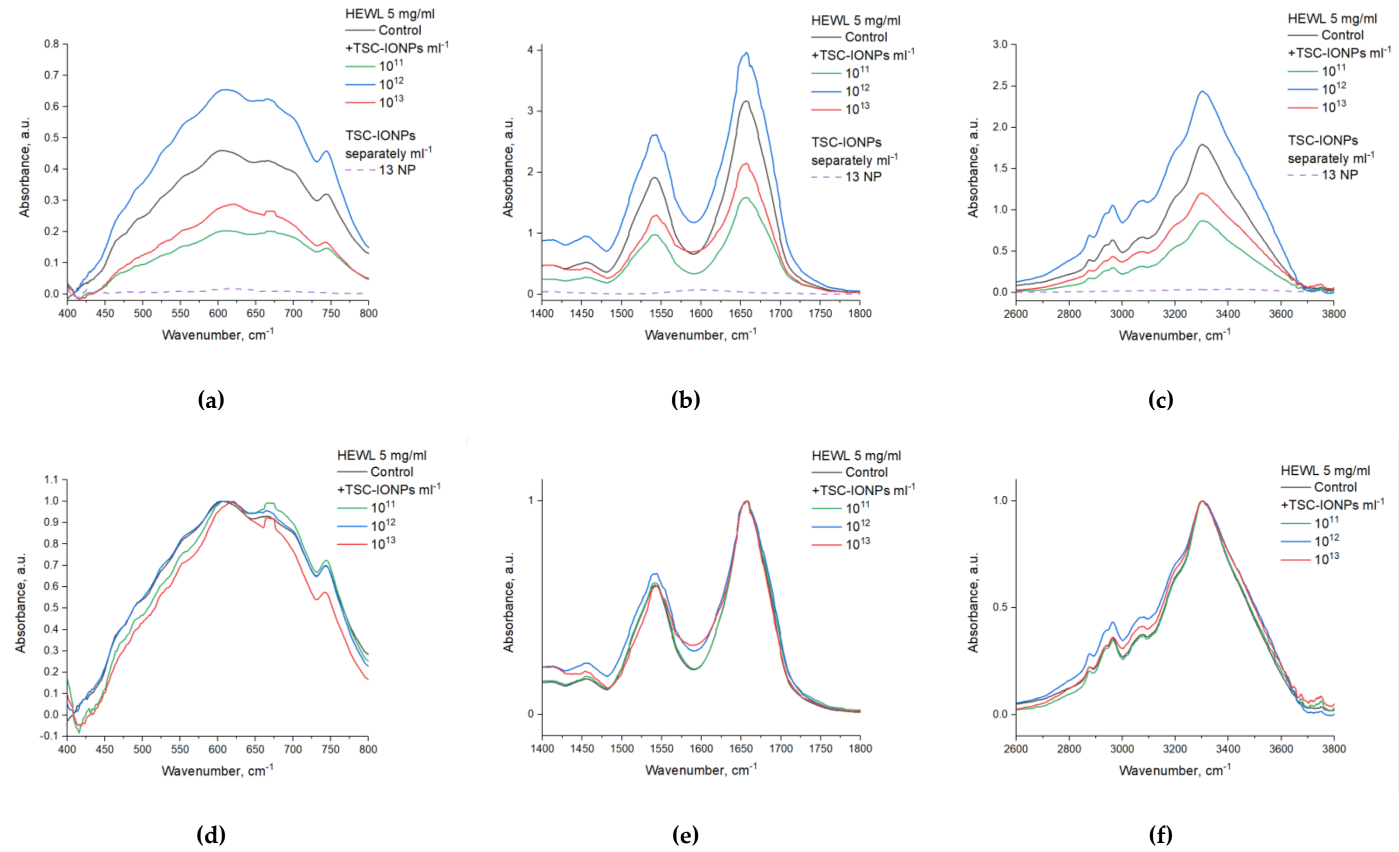

Figure S16 FTIR absorption spectrum for control HEWL solution (5 mg/ml) and with the addition of TSC-IONPs 10^{11}, 10^{12}, and 10^{13} ml^{-1}. Original measurements (**a**)–(**c**), normalized measurements (**d**)–(**f**).

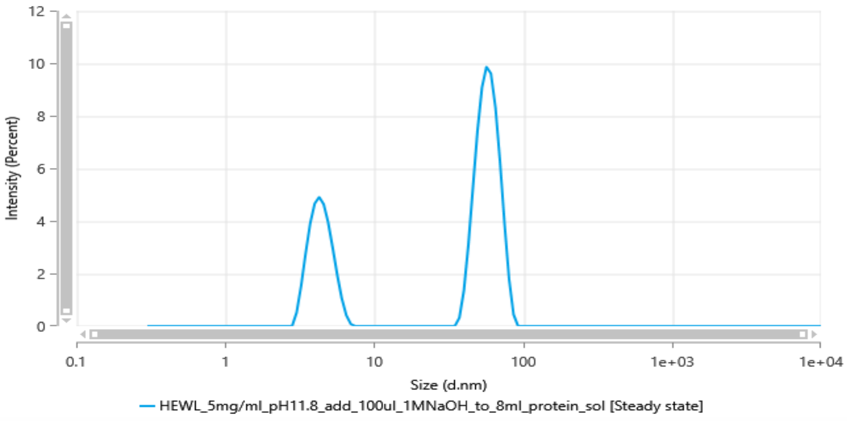

Figure S17 Distribution (weighted by intensity) of hydrodynamic diameters for molecules and aggregates for HEWL (5 mg/ml) with the addition of NaOH (pH = 11.8).
